# BRCore: an R package implementing flexible selection of core taxa using contribution to Bray-Curtis dissimilarity and neutral model fitting

**DOI:** 10.1128/mra.00251-26

**Published:** 2026-06-15

**Authors:** Bolívar Aponte Rolón, Brandon Kristy, Ashley Shade, Nejc Stopnisek, Sarah L. Lebeis, Adina Howe, Gian Maria Niccolò Benucci

**Affiliations:** 1 Department of Agricultural and Biosystems Engineering, Iowa State University 669136 , Ames, Iowa, USA; 2 Center for Advanced Bioenergy and Bioproducts Innovation, University of Illinois Urbana-Champaign 14589 , Urbana, Illinois, USA; 3 Department of Integrative Biology, Michigan State University 172705, East Lansing, Michigan, USA; 4 Great Lakes Bioenergy Research Center (GLBRC), East Lansing, Michigan, USA; 5 CNRS, INRAE, LEM, UMR 5557, UMR 1418, Université Lyon 1 27098, Villeurbanne, France; 6 Department for Microbiological Research, National Laboratory of Health, Environment and Food 68924, Maribor, Slovenia; 7 Department of Agricultural Ecology and Natural Resources, Agricultural Institute of Slovenia 54768 , Ljubljana, Slovenia; 8 Department of Plant, Soil, and Microbial Sciences, Michigan State University 242456, East Lansing, Michigan, USA; 9 Department of Microbiology, Immunology and Genetics, Michigan State University 220155, East Lansing, Michigan, USA; 10 Plant Resilience Institute, Michigan State University 3078 , East Lansing, Michigan, USA; University of Michigan, Ann Arbor, Michigan, USA

**Keywords:** microbiome statistics, community ecology, persistent taxa, ASV/OTU abundance-occupancy distributions, Bray-Curtis dissimilarity, Sloan neutral model

## Abstract

Identifying core taxa in microbial ecology highlights groups likely to participate in a broad range of potential ecological interactions. Here, we present BRCore, an R package to identify core taxa using abundance-occupancy distributions and beta-diversity contributions across ecological niches, and predict stochastic and deterministic taxa.

## ANNOUNCEMENT

Researchers define persistent microbiomes to identify abundant taxa, specifically those consistently present across samples, or enriched under distinct conditions. Core taxa selection is based on relative abundance and consistent detection, tailored to the research question, data set characteristics, and study system.

We introduce BRCore, a cross-platform R package compatible with Linux, macOS, and Windows, which implements the workflow proposed by Shade and Stopnisek (1) and incorporates additional rarefaction steps. The proposed workflow aims to identify persistent microbiomes using abundance-occupancy distributions and neutral community model fitting. Neutral models have previously been used to evaluate potential community assembly processes based on the underlying distributions of persistent taxa (1). Poor goodness-of-fit in neutral models has been used as evidence for deterministic selection (1–3). BRCore defines persistent taxa based on their contribution to beta-diversity, specifically Bray-Curtis dissimilarity (4), and provides neutral model testing, while accounting for features such as crop, site, or time for detection. This approach recognizes that high occupancy or abundance alone may not identify ecologically relevant taxa, and that persistent microbiomes may be transient and context-dependent across temporal and spatial gradients (1). Additionally, BRCore implements rarefaction of ASV/OTUs (i.e., repeatedly subsampling to a fixed depth without replacement) (5) and provides several visualization functions. BRCore requires R version ≥4.4 and depends on *phyloseq* (≥ 1.54.0), *dplyr* (≥ 1.1.4), *ggplot2* (≥ 4.0.1), *vegan* (≥ 2.7.2), and *rlang* (≥ 1.1.6).

**Fig 1:**
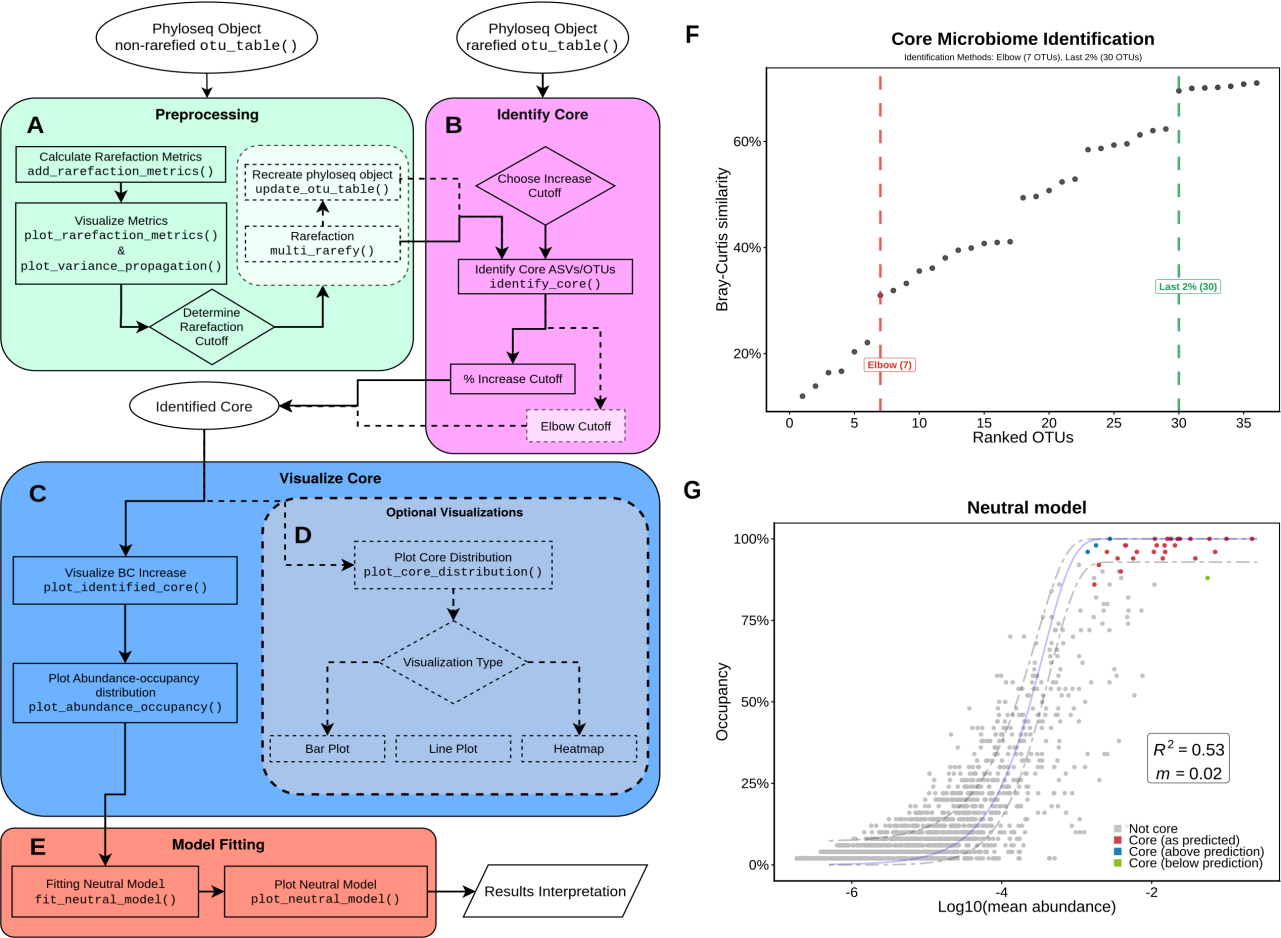
BRCore ASV/OTUs example pipeline. (**A**) Pre-rarefaction metrics plots are generated using add_rarefaction_metrics() and plot_rarefaction_metrics() to select optimal depth cutoff value based upon visuals (e.g., sample sequence distribution [observed and log10] and Good’s coverage value per-sample). The chosen rarefaction cutoff can be further investigated using plot_variance_propagation() to assess noise across rarefaction iterations and its impact on treatment group comparisons. Once a depth level is established, the multi_rarefy() and update_otu_table() functions perform rarefaction based on user-defined N iterations of resampling without replacement and chosen depth cutoff and incorporate one selected iteration into the otu_table() of the phyloseq object, respectively. (**B and C**) The identify_core() and plot_identified_core() identify and visualize (**F**) the core ASV/OTUs using both the *elbow* and *% increase* methods. (**D**) The plot_core_distribution() function produces a heatmap to visualize the relative occupancy of prevalent ASV/OTUs across a user-selected variable (i.e., “Crop” for the *bcse* data set). (**E**) The fit_neutral_model() and plot_neutral_model() functions fit and visualize the neutral models of ASV/OTUs abundance-occupancy distributions (**G**). The *R*^2^ is a standard r-squared statistic of goodness of fit (*R^2^ =* 1 *−* SS_err_*/*SS_total_), while *m* is the immigration parameter and represents the probability that a given individual in a sample originated from the metacommunity rather than from the local community (2, 3).

As a demonstration, we applied BRCore to the built-in “bcse” data set, which comprises leaf microbiome samples collected from 10 cropping systems at the Bioenergy Cropping System Experiment in Kellogg Biological Station (6) (Fig. 1). In the pre-processing step, we established the rarefaction depth cutoff using various diagnostics, including per-sample read depth plots and Good’s coverage (7) assessments (Fig. 1A), and determining noise propagation across rarefaction iterations (8). The data set was rarefied to 1,000 sequences per sample across three iterations. We computed Bray-Curtis dissimilarities for each iteration, and averaged these to identify core taxa. *Elbow* and *2% increase* cutoffs were applied and plotted (Fig. 1B through F); see Shade and Stopnisek (1) for details. Briefly, the *elbow* method identifies the point (*k* threshold) at which incremental gains begin to plateau and additional retained taxa yield diminishing returns, while the *2% increase* method maintains taxa until the additional gain from retaining one more taxon falls below a specified percentage (e.g., 2%). Neutral model goodness-of-fit statistics were calculated, including migration rate (*m*), coefficient of determination (*R*²), root mean square error (RMSE), Akaike information criterion (AIC), and Bayesian information criterion (BIC) for both binomial and Poisson fits; *R*² and *m* are shown in the plots (Fig. 1E and G).

The BRCore R package offers a reproducible, user-friendly, and flexible pipeline for persistent microbiome identification. It enables users to emphasize study-specific conditions or treatments and to interpret ecological niches meaningfully.

## Data Availability

BRCore is available on GitHub (https://github.com/germs-lab/BRCore), Zenodo (https://doi.org/10.5281/zenodo.18866205), and The Comprehensive R Archive Network (https://cran.r-project.org/package=BRCore).

## EDITOR'S NOTE

**Ed. note**: This article was handled by Pat Schloss, who acted as a guest editor in consultation with the editor in chief, Irene Newton. Dr. Schloss's role as the guest editor of this article is outside of his duties as the chair of the Journals Committee.
